# Sharing CD4+ T Cell Loss: When COVID-19 and HIV Collide on Immune System

**DOI:** 10.3389/fimmu.2020.596631

**Published:** 2020-12-15

**Authors:** Xiaorong Peng, Jing Ouyang, Stéphane Isnard, John Lin, Brandon Fombuena, Biao Zhu, Jean-Pierre Routy

**Affiliations:** ^1^ Infectious Diseases and Immunity in Global Health Program, Research Institute, McGill University Health Centre, Montréal, QC, Canada; ^2^ Chronic Viral Illness Service, McGill University Health Centre, Montréal, QC, Canada; ^3^ State Key Laboratory for Diagnosis and Treatment of Infectious Diseases, National Clinical Research Center for Infectious Diseases, Collaborative Innovation Center for Diagnosis and Treatment of Infectious Diseases, The First Affiliated Hospital, College of Medicine, Zhejiang University, Hangzhou, China; ^4^ Chongqing Public Health Medical Center, Chongqing, China; ^5^ CIHR Canadian HIV Trials Network, Vancouver, BC, Canada; ^6^ Division of Hematology, McGill University Health Centre, Montréal, QC, Canada

**Keywords:** COVID-19, HIV, CD4 exhaustion, cytokine storm, leaky gut

## Abstract

COVID-19 is a distinctive infection characterized by elevated inter-human transmission and presenting from absence of symptoms to severe cytokine storm that can lead to dismal prognosis. Like for HIV, lymphopenia and drastic reduction of CD4+ T cell counts in COVID-19 patients have been linked with poor clinical outcome. As CD4+ T cells play a critical role in orchestrating responses against viral infections, important lessons can be drawn by comparing T cell response in COVID-19 and in HIV infection and by studying HIV-infected patients who became infected by SARS-CoV-2. We critically reviewed host characteristics and hyper-inflammatory response in these two viral infections to have a better insight on the large difference in clinical outcome in persons being infected by SARS-CoV-2. The better understanding of mechanism of T cell dysfunction will contribute to the development of targeted therapy against severe COVID-19 and will help to rationally design vaccine involving T cell response for the long-term control of viral infection.

## Introduction

An outbreak of an unknown infectious pneumonia occurred in Wuhan, China, in December 2019 (1). The pathogen of the disease was quickly identified as a novel coronavirus coined severe acute respiratory syndrome coronavirus 2 (SARS-CoV-2), and the disease was named coronavirus disease-19 (COVID-19) by the WHO (2–4). The virus has since caused more than 48 million confirmed cases and over 1.2 million deaths worldwide by November, 2020 (5). The majority of individuals with COVID-19 have mild clinical presentation with or without flu-like symptoms including dry cough, fever, a runny nose, fatigue, muscle pain and diarrhea. Some cases can evolve into acute respiratory distress syndrome, septic shock, coagulation dysfunction, and multiorgan failure (1, 6, 7). The severity of the disease is influenced by factors such as older age, obesity and metabolic syndrome (8, 9). Acute infection with SARS-CoV-2 is associated with lymphopenia in approximately 80% of patients (6, 10–21). Furthermore, lymphopenia with the suppression of B, helper (CD4+) and cytotoxic (CD8+) T cell function, is an indicator of a poor clinical outcome (10–15, 17–19, 21–27). It is likely that lymphopenia delays viral clearance, favoring macrophage stimulation and the accompanying cytokine storm, leading to organ dysfunction (7, 15, 18, 19, 21, 23, 24, 26, 28, 29).

Apart from SARS-CoV-2, other viruses—including SARS coronavirus, measles virus, avian influenza virus H5N1, swine foot-and-mouth disease virus, respiratory syncytial virus and human immunodeficiency virus (HIV)—are associated with lymphopenia (30). Among them, HIV can cause an well-known lymphopenia-associated disease acquired immune deficiency syndrome (AIDS) (31). The acute phase of HIV infection is characterized by a substantial drop in peripheral CD4+ T cell counts, while during the chronic phase, a slower and persistent decline of these CD4+ T cells is associated with the development of AIDS. Antiretroviral therapy (ART) rapidly suppresses HIV replication, and the number of CD4+ T cell counts recovers, preventing AIDS. However, systemic immune activation persists in those people even after years of ART (32), and is characterized by increased proinflammatory mediators and low CD4/CD8 ratio (33), combined with exhausted and senescent T cells. Systemic immune activation is also associated with non-infectious comorbidities, such as cardiovascular diseases, neurocognitive disorders and cancers.

CD4+ T cells orchestrate the response to acute and chronic viral infections by coordinating the immune system. These cells activate multiple cells of the innate immune system, as well as B cells, cytotoxic CD8+ T cells, and non-immune cells. CD4+ T cells also play a key role for the establishment of long-term cellular and humoral antigen specific immunity, which is the basis of life-long protection for many viral infections and vaccines (34, 35).

Both HIV-1 and SARS-CoV-2 have distinct virological characteristics while sharing CD4+ T cell lymphopenia. In this review, we critically assessed the possible mechanisms and the potential influence of CD4**+** T cell lymphopenia in acute and chronic viral infections. We also discuss host characteristics and hyper-inflammatory response in these two dramatic viral infections and the impact of COVID-19 infection in people living with HIV (PLWH).

## The T Cell Dysregulation in PLWH and COVID-19

**Figure 1:**
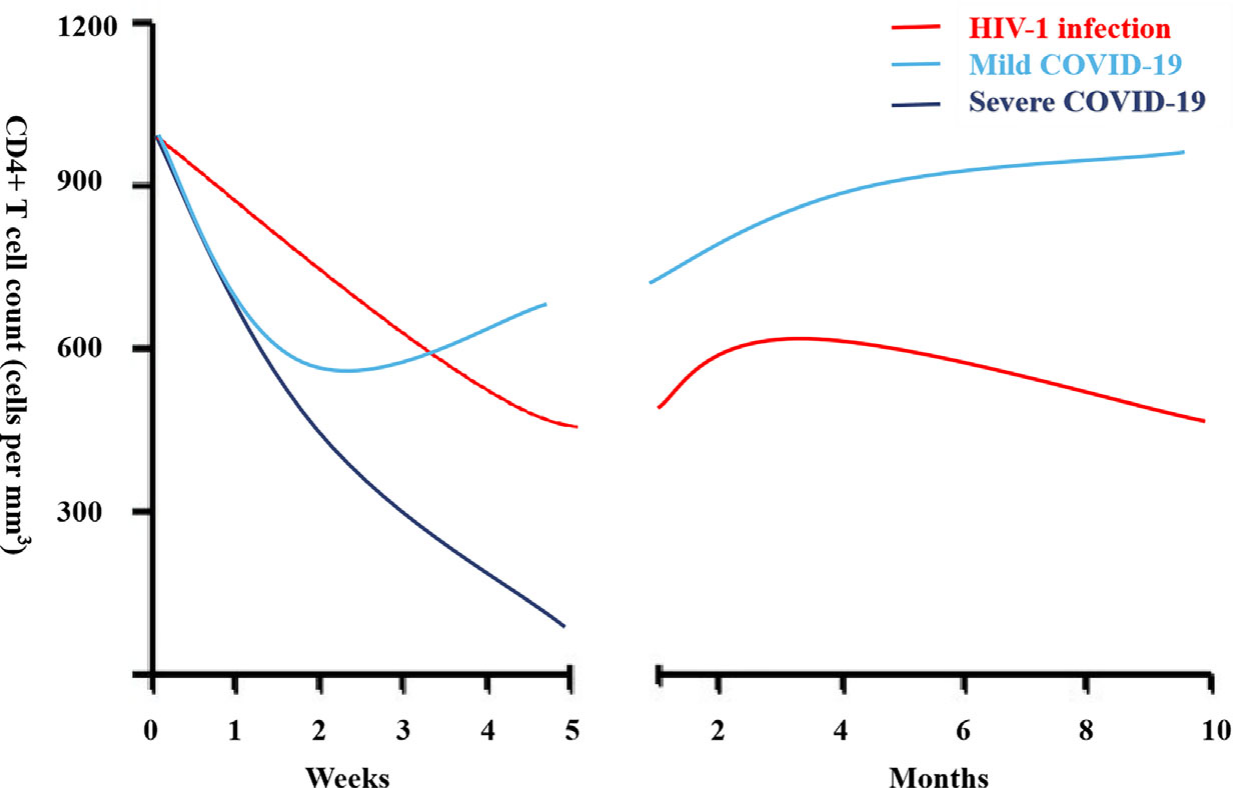
CD4+ lymphocyte count during acute infection in people living with HIV (PLWH) and coronavirus disease-19 (COVID-19).

The acute phase of HIV infection is characterized by a substantial drop in peripheral CD4+ T cell counts while in the chronic phase, a continued decline of CD4+ T cells is associated with the development of AIDS (**Figure 1**). In contrast, expansion of CD8+ T cells is observed which is driven mainly by an exhausted cytotoxic response toward HIV, leading to an inversed CD4/CD8 ratio. Despite ART, PLWH still present persistent immune activation and inflammation. The expressions of CD38 and HLA-DR as well as programmed death -1 (PD-1) are biomarkers of activated T cells, contributing to T cell exhaustion (36). Exhausted virus-specific CD4+ T cells express PD-1 at elevated levels correlating with disease progression, viral loads and reduced CD4+ T cell count (37).

Compared to healthy controls, in both acutely and chronically PLWH, the absolute number of regulatory T cells (Tregs) in the circulating blood is decreased, however the percentage of Tregs in chronic infection is increased (38, 39), further contributing to T cell dysfunction. Gut CD4+ T cells with a mucosa protective Th17 function are rapidly depleted (40) contributing to mucosal barrier dysfunction, leading to increase microbial translocation and systemic immune activation (41). Despite the decreased CD4+ T cell subgroup, both cell number and relative percentage of circulating T follicular helper (Tfh) cells increased in the blood during the chronic phase of HIV infection (42, 43). Tfh cells provide help to B cells in germinal center of secondary lymphoid organs and are central to the generation of efficient neutralizing and non-neutralizing antibody responses in HIV infection and will be essential in generating an effective vaccine (44). Expansion and altered features of HIV-specific and non-HIV specific circulating Tfh cells do not improve during ART and may be driven by persistent HIV antigen expression (45). Viral suppression by ART resulted with a reduction in the expression of genes associated with Tfh cells compared to viremic phase, which is accompanied by persistently low expression of genes associated with Th17 cells compared to persons who spontaneously control viremia (46).

Lymphocytopenia is a hallmark of patients with severe COVID-19 (6, 10–15, 17–19, 21) and is associated with poor clinical outcomes. The CD4+ lymphocyte count dynamic during mild and severe COVID-19 is shown in **Figure 1**. Helper CD4+ T cells are important in mediating protective humoral immunity by stimulating B cells to produce virus-specific antibodies. On the other hand, CD8+ T cells are responsible for the elimination of infected cells, mainly through the production of perforin and granzyme, and are key players in controlling different types of viruses through the secretion of cytokines. Both CD4+ and CD8+ T cell counts are reduced in severe COVID-19 (10–15, 17–19, 21–27). Similarly, reduced B cell counts are also observed in severe COVID-19 (14, 23). Moreover, within the CD4+ T cell subset, decreased numbers of effector memory T cells (CD45RO+) and Tregs (CD25+CD127low) were noted, while the proportion of naive T cells (CD45RA+) increased (16). The frequency of Tregs, which are responsible for the maintenance of immune homeostasis by suppressing activation and pro-inflammatory functions, was very low in severe cases. In addition, relative increased recirculation of activated CXCR5+PD-1^high^ CD4+ Tfh cells is observed in severe COVID-19.

**Figure 2:**
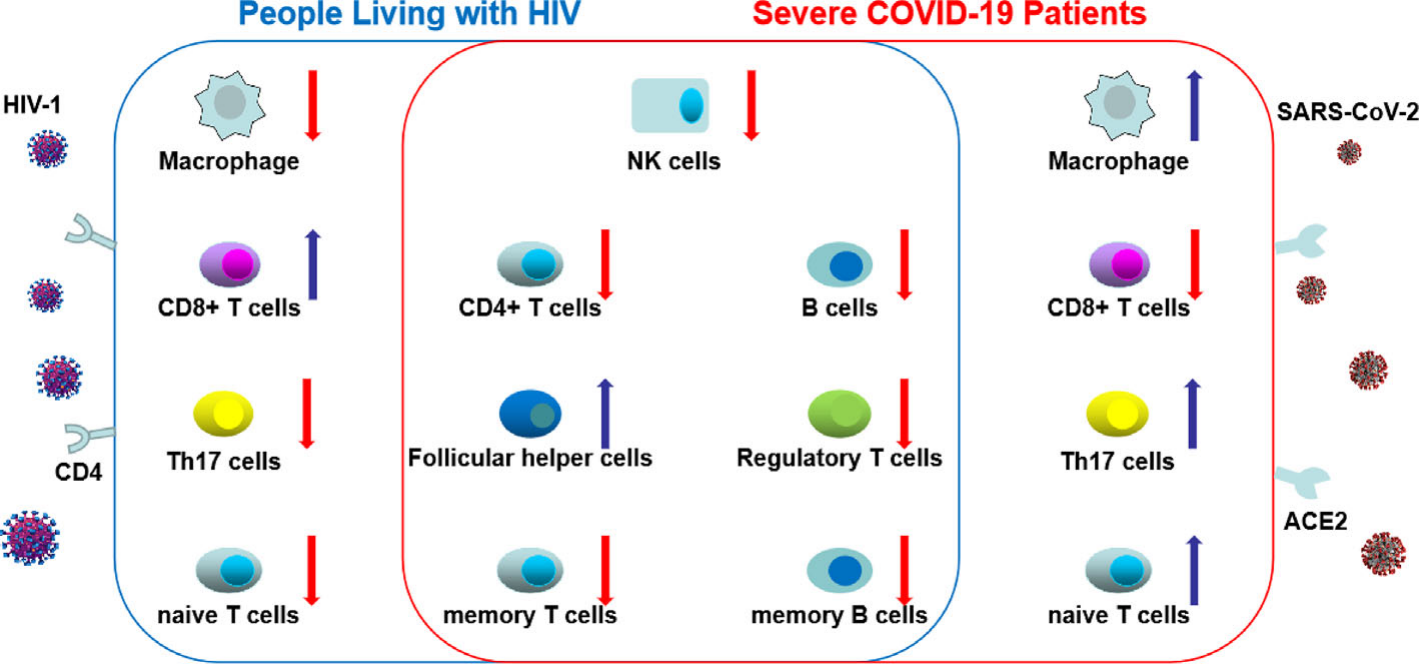
The changes of different peripheral blood cell types in HIV and severe coronavirus disease-19 (COVID-19). In COVID-19 and HIV infection, total count of natural killer cells, B cells, CD4+ T cells, regulatory T cells, memory T and B cells decrease, whereas the count of follicular helper cells increase. These common changes between HIV and COVID-19 were shown in the central circle. However, distinct changes were shown in total count of macrophage, CD8+ T cells, Th17 cells and naive T cells between people living with HIV (PLWH) and severe COVID-19. (The red arrow indicates a decrease in the number of cells; the blue arrow indicates an increase in the number of cells).

CD4+ T cells in COVID-19 are activated as characterized by expression of cellular markers like HLA-DR, CD25, CD38 and Ki-67 (47). T cell exhaustion based on increased inhibitory markers such as PD-1 and TIM-3 receptor on peripheral T cells has also been reported (47–49). Studies have demonstrated that decreases in polyfunctionality (multiple cytokine secretion) and cytotoxicity of T cells were correlated with disease progression (21, 49). Conversely to HIV, a study demonstrated an increase in the number of Th17 cells in the peripheral blood in COVID-19 patients (50). In hospitalized patients compared to non-hospitalized patients, Mathew *et al.* found increased proportion of cytotoxic follicular helper cells and cytotoxic T helper cells responding to SARS-CoV-2 and reduced proportion of SARS-CoV-2-reactive Treg cells (47). Elevated SARS-CoV-2-specific CD4+ and CD8+ T cells were each associated with milder disease, fostering important roles for both CD4+ and CD8+ T cells in protective immunity in COVID-19 (51). Furthermore, absence of these virus-specific cells leads to uncoordinated antigen-specific immune responses and failure to control COVID-19, predominantly in older individuals with low naïve CD4+ T cells. Similarly, PLWH are not able to mount an effective HIV-specific CD4+ and CD8+ T cell responses with the exception of HIV controllers who can maintain undetectable or low levels of viremia despite not being on ART (46, 52, 53). Features of different peripheral blood cell types in PLWH and severe COVID-19 are shown in **Figure 2**.

## Leaky Gut

Depletion of gut CD4+ T cells will be followed by disruption of the tight junctions, and cell death of intestinal epithelium. Epithelial gut damage leads to both an imbalance of the intestinal microbiota composition (dysbiosis) and the release of bacterial products in the circulation (microbial translocation), participating in chronic immune activation and inflammation (54, 55).

Apart from relevant metabolic functions for the host homeostasis, the gut microbiota exerts protective actions against pathogenic colonization of bacteria and viruses, which could be at least partially attributed to their role in educating and strengthening the immune system (56). The triad gut microbiota dysbiosis–immune hyper-response–inflammation is involved in both HIV and COVID-19 pathogenesis (57).

Within a few weeks of HIV infection, the virus begins a massive assault on the gut, which undergoes a significant depletion of CD4+ T cells with Th17 function (58). PLWH have an altered microbiota composition with an increase of pro-inflammatory and potentially pathogenic bacteria as well as a decrease of beneficial bacteria (59, 60). Gut damage allows microbial translocation, a cause of systemic immune activation in chronic HIV which is usually determined by measuring plasma levels of markers of microbial translocation such as lipopolysaccharide (LPS) and (1→3)-β-D-Glucan (BDG), all of which are elevated in PLWH, even those on ART (61, 62). Previous studies have shown that LPS and BDG were associated with disease progression, lower CD4+ T cell count, and induce immune activation (61–63).

Over 60% of patients with COVID-19 report evidence of gastrointestinal symptoms, such as diarrhea, nausea and vomiting (64). There is direct evidence that SARS-CoV-2 can replicate in intestinal cells (65). Moreover, many viral infections, including influenza, drive changes in the gut and lung microbiota with viral-mediated changes in the gut including dysbiosis and increased permeability (66). Indeed, recent studies found some differences in gut microbial features and related metabolites in SARS-CoV-2 infection (67). More attention should be directed to gut dysbiosis and microbial translocation in the contribution to severe COVID-19.

## Hyper-Inflammation in HIV Infection and COVID-19

Examination of plasma cytokines of acute HIV infection revealed that interferon (IFN)-α was the first cytokine to be increased within a few days after detection of viremia, followed by tumor necrosis factor α (TNF-α), IFN-γ, and interleukin (IL)-12 (68). Initiation of ART during Fiebig stages I-II can abrogate the HIV-induced cytokine storm (69). Elevation of IFN-α, IFN-γ, monocyte chemoattractant protein (MCP)-1, soluble IL-2 receptor (sCD25), IL-6 and IL-8 was seen in chronically-infected untreated individuals (63, 70, 71). Initiation of ART significantly reduces plasma levels of inflammatory cytokines, markers of inflammation and monocyte activation, without normalization compared to HIV-uninfected individuals (72).

Similarly, in COVID-19 patients, elevation of inflammatory cytokines was also observed. In severe cases, elevations of TNF-α, IFN-γ, IL-2R, IL-6, IL-8, and IL-10 were detected (7, 15, 18, 19, 21, 23, 24, 26, 28, 29). However, a highly impaired interferon (IFN) type I response was observed, characterized by no IFN-β and low IFN-α production and activity (73). Furthermore, studies have found increased production of proinflammatory cytokines and chemokines, IL-2, IL-7, IL-10, granulocyte colony-stimulating factor (G-CSF), CXCL-10/IP-10, TNF-α and macrophage inflammatory protein (MIP)-1α in intensive care unit (ICU) patients compared with non-ICU patients (7). In addition, IL-6 levels were considered as a biomarker of disease severity and mortality (28, 29) and ongoing clinical trials are assessing IL-6 blockade to improve outcome in COVID-19 patients (74).

A pre-existed low-level inflammation and leaky gut in type 2 diabetes mellitus (T2DM) may be associated with higher COVID-19 mortality (75, 76). Retrospective studies have shown a reduction in mortality in metformin users compared with non-users among patients with T2DM hospitalized for COVID-19 (77). The potential effects of metformin in COVID-19 could be through inhibition of the mTOR pathway and prevention of immune hyperactivation (78). Reduced production of cytokines such as TNF-α and IL-6 was seen in metformin-treated patients (79). Furthermore, metformin may also reduce inflammation by altering the composition of gut microbiota (80, 81). A retrospective cohort study on PLWH with diabetes mellitus showed that Metformin use was associated with improved CD4 recovery (82). Whether metformin could be a potential treatment strategy for CD4+ T cells lymphopenia in COVID-19 need further investigation.

## Comorbidities in PLWH and COVID-19

Although ART reduced the risk of developing AIDS (83), it does not normalize inflammation that is associated with risk of non-AIDS comorbidities, including cardiovascular and metabolic diseases and neurocognitive dysfunctions (84–86).

In COVID-19, direct viral attack and systemic hyper-inflammation can cause dysfunction of several organs. Postmortem analyses showed that the main damage occurred in the lungs, to the alveolar epithelial cells, hyaline membrane formation, and hyperplasia of type II pneumocytes, all components of diffuse alveolar damage (87, 88). Nearly 20% of patients hospitalized for COVID-19 in Wuhan, China showed evidence of cardiac injury (89, 90). More than half of COVID-19 patients hospitalized had elevated levels of enzymes indicating injury to the liver (91). In a case series of 214 patients with COVID-19, neurologic symptoms were seen in 36.4% of patients which included acute cerebrovascular events, impaired consciousness, and muscle injury (92).

## Mechanisms of CD4+ T Cell Lymphopenia

**Figure 3:**
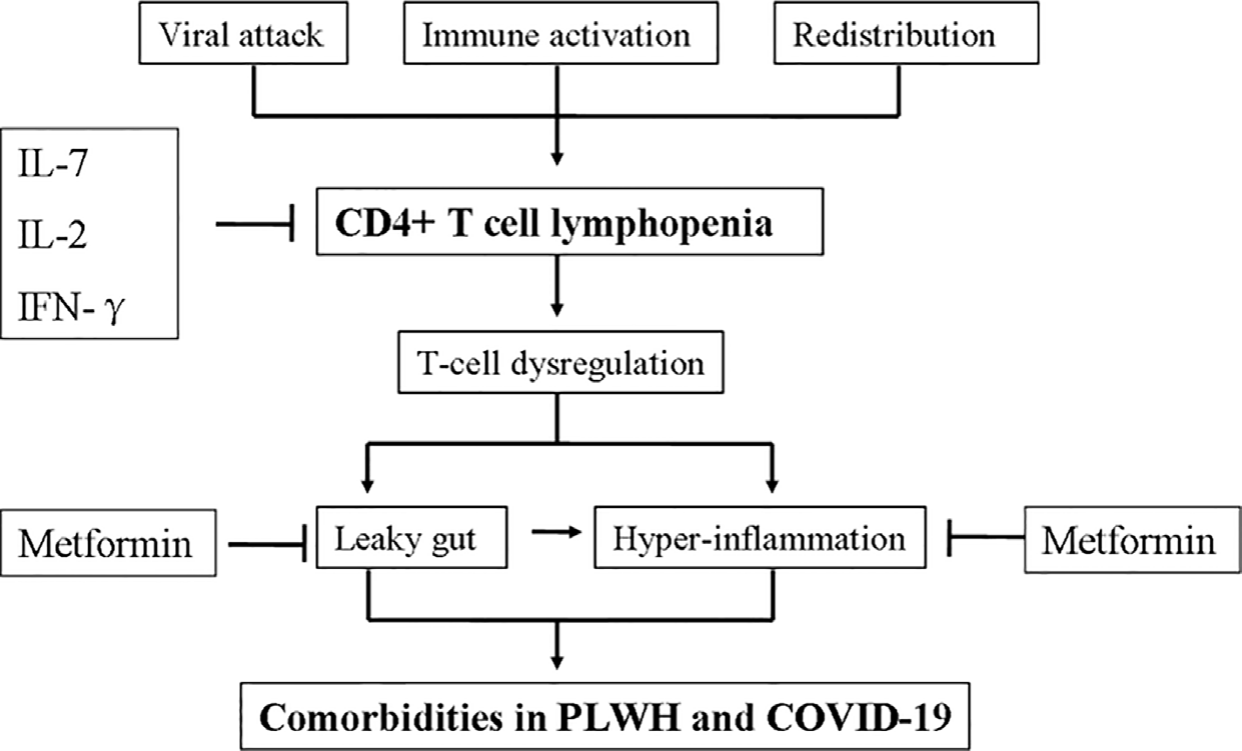
Potential mechanism and consequence of CD4+ lymphopenia in people living with HIV (PLWH) and coronavirus disease-19 (COVID-19).

The thymus supports T cell differentiation from T progenitor cells, which differentiate from hematopoietic stem cells in bone marrow, and selects mature CD3+ CD4+ and CD3+ CD8+ thymocytes (41). Quantitative estimates indicate that healthy young (<30 year old) adults harbor about 2.2×10^11^ mature CD4+ T cells (93). Most CD3+CD4+ and CD3+CD8+ T cells reside in peripheral lymphoid organs where T and B cell responses are coordinated by antigen-presenting cells (APC). CD4+ T cell numbers are kept constant in the human body by homeostatic mechanisms including IL-7 (41). Total CD4+ T cells may be depleted due to cell death, shortened half-life or impaired production. In addition, the proportion of circulating CD4+ cells may decrease through lymphoid tissue redistribution at sites of inflammation. A number of dynamic models have been put forth explaining HIV-mediated depletion of CD4+ T cells (94, 95). However, CD4+ lymphopenia is poorly understood in COVID-19. Potential mechanisms and consequences of CD4+ lymphopenia in PLWH and COVID-19 are shown in **Figure 3**.

### Direct Attacks on CD4+ T Cells

Early experiments done with laboratory-adapted HIV isolates in tissue culture revealed a cytopathic virus with high tropism for CD4+ T cells (96). There is a homeostatic response by which the loss of CD4+ T cells due to HIV infection is counteracted by production of T cells; however, this balance is ultimately disrupted once the production of T cells in response to homeostasis is exhausted. This has been substantiated by quantitative image analysis of decreased numbers of CD4+ T cells and increased levels of cellular proliferation and apoptosis in PLWH (97, 98). However, evidence showed that HIV pathogenesis cannot be solely explained by the direct viral killing hypothesis as uninfected CD4+ T cells have a shortened half-life by cellular viral contact affecting IL-7 signalization (99). Another explanation is phospholipase A2 group IB (PLA2G1B) which synergizes with the HIV gp41 envelope protein and targets the CD4+ T cell surface, leading to CD4+ T cell unresponsiveness (anergy) (100).

The question arises whether SARS-CoV-2, like HIV, can directly decrease CD4+ T cell count. ACE2 (angiotensin-converting enzyme 2) is the SARS-CoV-2 internalization receptor (101), in concert with the host’s TMPRSS2 (transmembrane protease serine 2) membrane protease that primes the coronavirus spike S protein to facilitate its cell entry (102). ACE2 and TMPRSS2 are co-expressed in lung, heart, liver, kidney, neurons and immune cells (103). Immune cells could potentially be infected by SARS-CoV-2, as in the case of SARS-CoV (104), with both viruses sharing the same receptor ACE2 (102). Studies showed that SARS-CoV can infect 50% of lymphocytes in the circulation (105), resulting in cell death by apoptosis, necrosis, or pyroptosis (106, 107). Furthermore, under the influence of SARS-CoV, the germinal center regressed, and both T and B lymphocytes are depleted (108). Extensive cell death of lymphocytes was observed in an autopsy study of spleens and hilar lymph nodes of six patients with COVID-19. However, the direct evidence of whether SARS-CoV-2 infects T cells is still lacking.

### Immune Activation and T Cell Death

Previous studies proposed that activated CD4+ T cells have a very short life span due to activation-induced cell death or apoptosis (109). In HIV infection, the activation of CD4+ T cells is driven by the antigenic stimulus by HIV proteins (110) and in part by antigen-independent mechanisms through the production of inflammatory cytokines. Continuous hyperactivation of T cells may lead to accelerated consumption of naïve T cells through apoptosis or differentiation toward a memory phenotype.

Elevation of inflammatory cytokines and cytokine storm was observed in COVID-19 patients. Previous studies showed that a number of inhibitory cytokines are released by infected lung macrophages or epithelial cells. These cytokines include TNF-α which causes T cell apoptosis (111), IL-10 which is known to prevent T cell proliferation (112), and type-I IFN which regulates lymphocyte recirculation (113). Whether these inflammatory cytokines contribute to the loss of CD4+ T cell needs further investigation.

### Redistribution of CD4+ T Cells

Circulating CD4+ T cell counts are most studied due to their ease of access. However, CD4+ T cell in the blood compartment does not always reflect the composition of lymphoid organs or infected sites where CD4+ T cells are recruited. Hence, CD4+ T cell lymphopenia could be a reflection of CD4+ T cell redistribution throughout the body.

Some evidence from simian immunodeficiency virus (SIV) macaque models indicates CD4+ T cell redistribution from the peripheral blood to lymph nodes and the gut (114). When blood levels of CD4+ lymphocytes begin to drop significantly, these cells often increase in number in the lymph nodes (115). This suggests that the loss of CD4+ T cells in the blood can in part be explained by an enhanced homing of CD4+ lymphocytes into the lymph nodes. Furthermore, CD62L, the receptor for lymph node homing, could be unregulated after infection with HIV (116). After the initiation of effective antiretroviral therapy, decreased levels of adhesion molecules like VCAM-1 and ICAM-1, which mediate lymphocyte sequestration into lymphoid tissue, were associated a rapid increased of CD4 T cells and decreased LN size (117).

SARS-CoV-2 prefentially infects and destroys alveolar epithelial cells that may in turn trigger the production or the overproduction by macrophages of pro-inflammatory cytokines and chemokines (including interleukin-6 (IL-6), IL-8, CXCL10/IP-10, CCL3/MIP1α, CCL4/MIP1β) (118). Secretion of such cytokines and chemokines attracts immune cells, notably monocytes and T lymphocytes, from the blood into the infected site, which may explain the circulating lymphopenia. Additionally, the first autopsy of a patient with COVID-19 revealed an accumulation of mononuclear cells (monocytes and T cells) in the lungs, coupled with low levels of hyperactive T cells in the peripheral blood (88). Furthermore, anti-IL-6 immediately reversed lymphopenia favoring tissue redistribution in patients having multicentric Castleman disease, a condition characterized by an enhanced level of IL-6 (119). Animal models and future clinical trials will help decipher the mechanism responsible for SARS-CoV-2 associated lymphopenia.

## When HIV Meets COVID-19

Several case reports assessed the influence of COVID-19 in PLWH (120–123). In a case series of 33 PLWH patients with COVID-19, three out of 32 patients with documented outcome died (9%). However, 91% of the patients recovered and 76% have been classified as mild cases, indicating that there is no excess morbidity and mortality among PLWH with symptomatic COVID-19 compared to COVID-19 HIV-negative patients (123). In a study in Wuhan, there were 8 COVID-19 out of 1174 investigated HIV/AIDS patients. The authors reported absence of influence of sex, CD4+ T cells counts, HIV viral load, or ART regimen associated with the occurrence of COVID-19, only older age was associated with COVID-19 infection (124).

## Hypotheses for the Non-Influence of HIV Infection in COVID-19 Disease

A compromised immune system with a lower CD4+ T cells counts and elevated interferon levels in HIV infection might reduce clinical symptoms of COVID-19. There is a hypothesis that a lower active immune status might protect the human body from a virus-induced cytokine storm, such as SARS and MERS (125).

Some ART medications (lopinavir/ritonavir, ritonavir, darunavir, and dolutegravir), were screened for anti-SARS-CoV-2 replication activity and were initially used to treat COVID-19 (126). However, clinical trials using lopinavir/ritonavir, a protease inhibitor that could suppress SARS-CoV-2 replication *in vitro*, had no impact on COVID-19 outcome (127). Another drug is tenofovir (TDF), a nucleoside analog of remdesivir, which can inhibit SARS-CoV-2 RNA-dependent RNA polymerase (RdRp) activity *in vitro* and shorten the time to recovery in adults who were hospitalized with COVID-19 and had evidence of lower respiratory tract infection (128).

In a cohort study with 77 590 HIV-positive persons receiving ART, the result showed that HIV-positive patients receiving TDF/Emtricitabine (FTC) had a lower risk for COVID-19 and related hospitalization than those receiving other therapies (129). These findings warrant further investigation in healthy individuals taking these two drugs for HIV preexposure prophylaxis studies and randomized trials in persons with and without HIV.

## Potential Treatment of CD4+ T Cells Lymphopenia

### IL-7

IL-7 levels are known to be inversely correlated with CD4+ T cell counts in patients with HIV/AIDS, and is likely associated with a homeostatic response (130). IL-7 is essential to B and T cell lymphopoiesis in the bone marrow. Clinical studies showed that recombinant IL-7 treatment increased the number of naive and memory CD4+ and CD8+ T cells while conserving T cell functions (131, 132). Several clinical trials are currently under way to evaluate the efficacy of IL-7 to improve clinical outcomes in lymphopenic patients with COVID-19 (NCT04407689, NCT04379076, NCT04442178 and NCT04442178).

### IL-2 and IFN-γ

IL-2 is a potent mitogen and growth factor in antigen-stimulated CD4+ T cells (133). IL-2 has been studied in HIV and has been shown to increase CD4+ T cells counts (134, 135). IL-2 levels in the peripheral blood were increased in severe COVID-19 cases compared to mild cases (7, 15, 18, 19, 21, 23, 24, 26, 28, 29). Whether IL-2 can be used to improve CD4+ T cell lymphopenia in COVID-19 patients should be carefully considered. The efficacy of low-dose IL-2 administration is under evaluation in patients with SARS-CoV2-related acute respiratory distress syndrome in a randomized controlled trial (NCT04357444).

## Limitations

There are still some knowledge gaps about CD4+ T cell loss in PLWH and COVID-19. Firstly, the dynamic of change in CD4+ T cell is difficult to be compared, especially as HIV induces both an acute and chronic disease state. Secondly, the data in COVID-19 are limited. New studies need to be conducted to learn more about this new disease, and lessons from studies on HIV infection and care of PLWH could definitely help designing new therapeutic tools.

## Conclusion

Both HIV-1 and SARS-CoV-2 infection share CD4+ T cell loss in association with disease outcome and immunodeficiency. Direct attacks on CD4+ T cells, immune activation and redistribution of CD4+ T cell are contributing mechanisms in very different proportion for CD4+ T cell lymphopenia in both diseases. During the period of immunodeficiency, systemic inflammation could be fueled by leaky gut and lead to severe complications. However, when HIV meets COVID-19, no increase in the occurrence of COVID-19 and no excess morbidity and mortality among PLWH with symptomatic COVID-19 has been reported. IL-7 and IL-2 were previously used to increase CD4+ T cell counts in HIV-1 infection, however, no improvement in their function were reported. Despite this, the short-term effect for COVID-19 is under investigation. As CD4+ T cells orchestrate immune responses, proper CD4+ T cell function is required for effective vaccine responses. Hence, anti-SARS-CoV-2 antibodies and CD4 responses should be studied in order to develop long-term efficiency vaccine formulation. Overall, experience in HIV clinical management and past clinical trials represent a special use case for innovative studies aiming at increasing CD4+ T cell function and reducing COVID-19 morbidity.

## Author Contributions

XP wrote the first draft of the manuscript. JO, SI, JL, BF, and BZ provided critical revision of the manuscript. J-PR conceived and designed the manuscript. All authors contributed to the article and approved the submitted version.

## Funding

This work was funded by the China Scholarship Council (No.201906325018), the Canadian Institutes of Health Research (CIHR; grants MOP 103230 and PTJ 166049), the Vaccines & Immunotherapy Core of the CIHR Canadian HIV Trials Network (CTN, grant CTN 257), the CIHR-funded Canadian HIV Cure Enterprise (CanCURE) Team Grant HB2-164064, This work was also supported by the Fonds de la Recherche Québec-Santé (FRQ-S): Réseau SIDA/Maladies infectieuses and Thérapie cellulaire. JO is supported by the Chinese National Science and Technology Major Project during the 13th Five-Year Plan (No. 2018ZX10302104). SI is supported by a Fond de Recherche Québec Santé fellowship and a CIHR/CTN Postdoctoral Fellowship Award. JP-R is the holder of the Louis Lowenstein Chair in Hematology and Oncology, McGill University and William Turner award holder from the McGill University Health Centre.

## Conflict of Interest

The authors declare that the research was conducted in the absence of any commercial or financial relationships that could be construed as a potential conflict of interest.
